# Orthogonal Relations and Color Constancy in Dichromatic Colorblindness

**DOI:** 10.1371/journal.pone.0107035

**Published:** 2014-09-11

**Authors:** Ralph W. Pridmore

**Affiliations:** Department of Cognitive Science, Macquarie University, Sydney, NSW, Australia; Lund University, Sweden

## Abstract

This paper employs uniform color space to analyze relations in dichromacy (protanopia, deuteranopia, tritanopia). Fifty percent or less of dichromats represent the classical reduction form of trichromacy, where one of three cones is inoperative but normal trichromatic color mixture such as complementary colors (pairs that mix white) are accepted by the dichromat, whose data can thus be plotted to CIE chromaticity spaces. The remaining dichromats comprise many and varied more-complex gene arrays from mutations, recombinations, etc. Though perhaps a minority, the three reductionist types provide a simple standard, in genotype and phenotype, to which the more complex remainder may be compared. Here, previously published data on dichromacy are plotted and analyzed in CIELUV uniform color space to find spatial relations in terms of color appearance space (e.g., hue angle). Traditional residual (seen) hues for protanopia and deuteranopia (both red–green colorblindness) are yellow and blue, but analysis indicates the protanopic residual hues are more greenish yellow and reddish blue than in tradition. Results for three illuminants (D65, D50, B) imply four principles in the spatial structure of dichromacy: (1) complementarity of confusion hue pairs and of residual hue pairs; (2) orthogonality of confusion locus and residual hues locus at their intersection with the white point, in each dichromatic type; (3) orthogonality of protanopic and tritanopic confusion loci; and (4) inverse relations between protanopic and tritanopic systems generally, such that one's confusion hues are the other's residual hues. Two of the three dichromatic systems do not represent components of normal trichromatic vision as sometimes thought but are quite different. Wavelength shifts between illuminants demonstrate chromatic adaptation correlates exactly with that in trichromatic vision. In theory these results clarify relations in and between types of dichromacy. They also apply in Munsell and CIELAB color spaces but inexactly to the degree they employ inexact complementarity.

## Introduction

Normal color vision is trichromatic for 2-degree visual fields, requiring mixtures of three primary color stimuli to match all colors. But some 2.4% of humans (99.7% of them male) are dichromats who require mixtures of only two primary color stimuli to match all colors [1], [2]. The most common forms are protanopia and deuteranopia (each some 1.2% of humans), also known as red-green blindness. Such dichromats see only two hues, blue and yellow. John Dalton published the first scientific paper on dichromacy in 1798 [3] after realizing his own red-green color blindness. A third and very rare type of dichromacy is tritanopia (known as yellow-blue blindness). All forms of dichromacy result from complete or partial loss of function of one of the three cones (short, medium, and long wavelength, S,M, L) and are usually congenital [1], [4], [5], caused by alterations in the opsin gene array encoding the photopigment. A lesser form of deficient vision, intermediate to normal trichromatic vision and dichromacy, is anomalous trichromacy (e.g., protoanomalous trichromacy); this form is not treated in this article.

**Figure 1:**
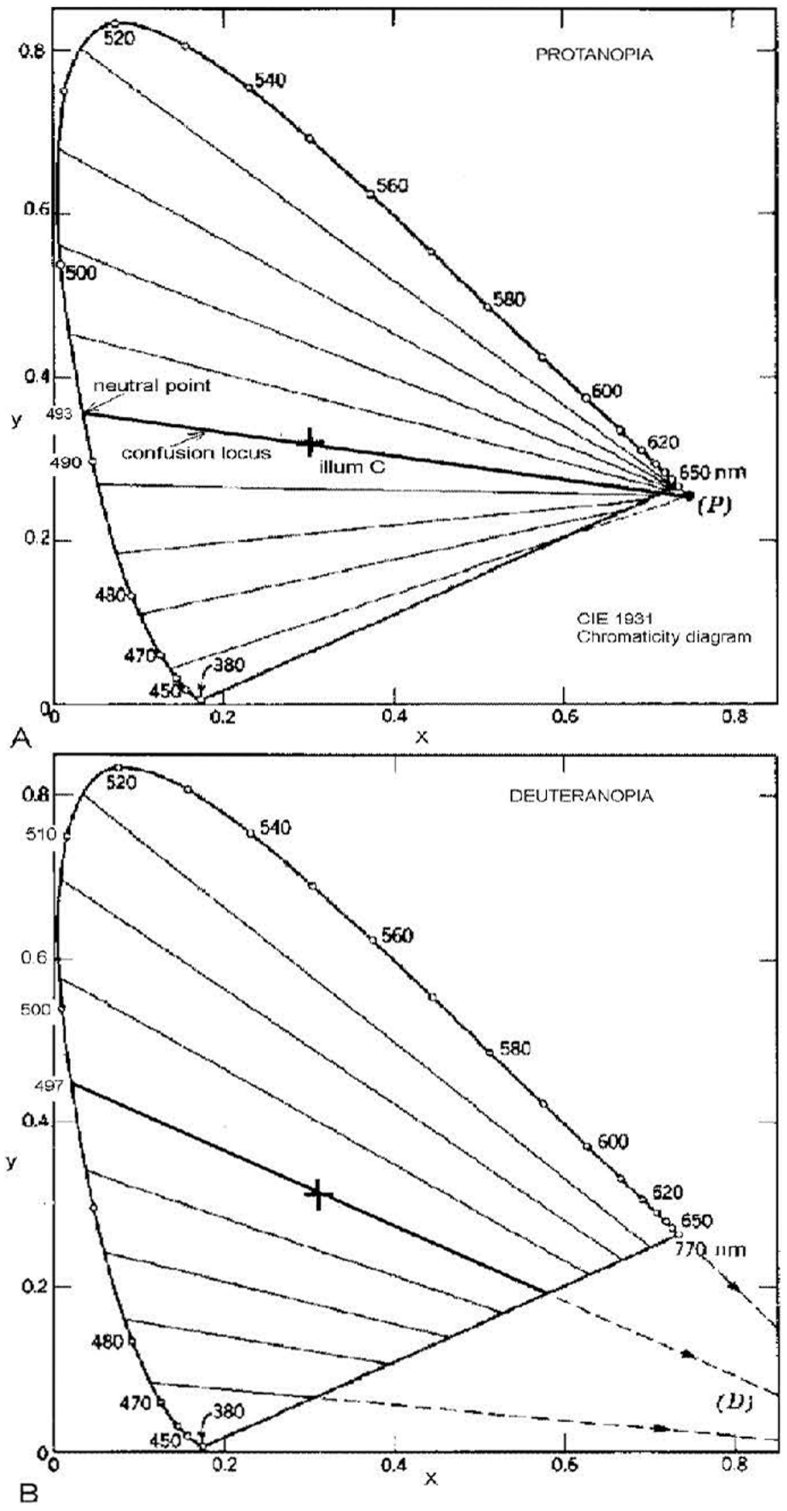
CIE 1931 chromaticity diagrams showing confusion loci for illuminant C with main confusion locus in heavy black line. A. For protanopia. Neutral point and confusion point P are labeled. B. For deuteranopia. Confusion point D is labeled. Redrawn from Judd [25].

Protanopia and deuteranopia are usually sex-linked conditions causing loss or deficient operation of either the L or M cones respectively. In protanopia and deuteranopia the genes that produce the photopigments in the cones are carried on the X chromosome, of which males have only one and females two. A functional gene on any one X chromosome is sufficient to yield the necessary photopigment. Hence, the deficiency will be expressed in males with a higher probability than in females [4], [5]. Protanopia and deuteranopia involve loss of discrimination of middle and long wavelengths (bluish-green and red hues) of the spectrum and some of the nonspectral or purple region, as shown in Figure 1.

Figure 1 features the Commission Internationale de l'Eclairage (CIE) 1931 chromaticity diagram here showing confusion loci for protanopia and deuteranopia. All colors on a confusion locus are confused by the relevant dichromat. The heavy black central line is the main confusion locus, from the neutral point on the spectrum locus (curved line representing the boundary of real colors) through the white or illuminant point to the confusion point on or near the purple line. This entire locus appears achromatic (neutral) to the dichromat. Other confusion loci, to either side of the main locus, indicate varying saturations of one of the two residual (seen) hues, blue or yellow.

**Figure 2:**
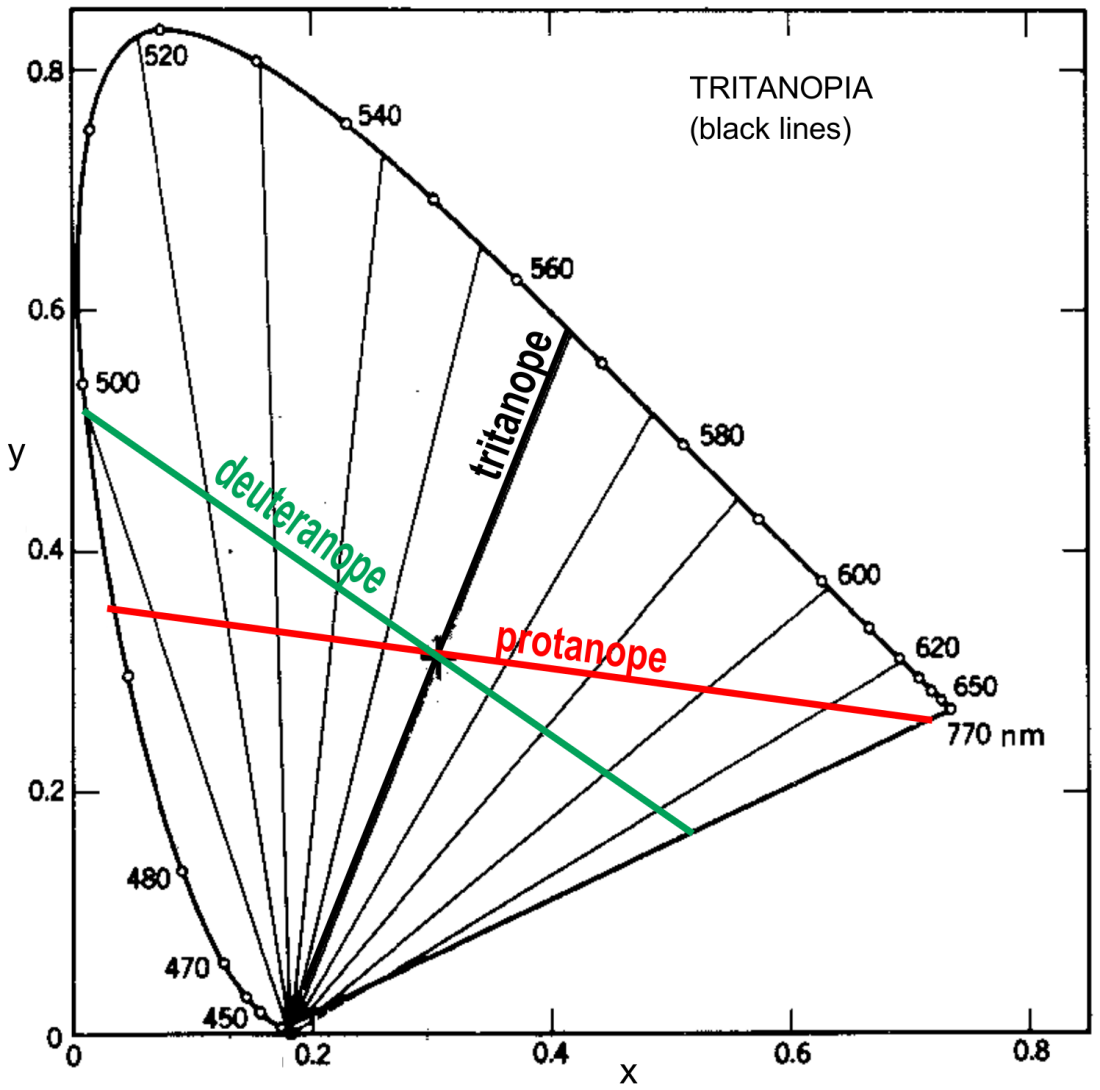
As for Figure 1 but for tritanopia (black lines). Main confusion loci from Figures A (red line) and B (green line) are superimposed. Redrawn from Judd [25].

Figure 2 shows confusion loci for tritanopia, called yellow-blue blind but also confusing blue-green hues and yellow–red hues. Tritanopes see mainly bluish-green (cyan) and red hues. Also shown are the main confusion loci from Figures 1A and 1B to show the interrelationship of all three confusion loci. Tritanopia is usually congenital but unlike protan and deuteran defects, it is autosomal (linked to chromosone 7 rather than X) and hence this form of colorblindness is not sex-linked. It occurs only in about 0.002% of the population [1], [4], [5], thought to be equally male and female, and reflects loss of S cone function, completely or partially.

Protanopia and deuteranopia were long thought to represent reduced forms of trichromacy, where one of the three cone pigments was lacking and the other two were normal. Dichromats were expected, and usually found, to accept the color matches of color normals, such as the white mixed from complementary colors. Assuming this reduced form of trichromatic vision was linear, even as an approximation, color matching functions (and color mixture) for each of the three types of dichromacy were predicted from normal trichromatic color mixture/ matching functions [1], [2]. This reductionist view was widely accepted till about the turn of the century but the situation is now known to be more complex [1], [2], [5], [6], [7], including polymorphism such as M and L cone alternative gene alleles which can shift the cone sensitivity peaks some 3 or 4 nm. It is now clear the true reduction dichromat is just one genotype in each type of dichromacy, and a large number of other more complex genotypes exist (from mutations, recombinations, polymorphism etc) causing a plethora of varieties especially of protanopia and deuteranopia [8].

Amongst all these varieties, the reductionist forms of protanopia, deuteranopia and tritanopia stand out as simple standards to which the more complex variations may be compared, related and understood. Although the reduction form of dichromacy exists in possibly less than half of all dichromats [4], [5], it probably represents the most numerous single genotype in each type of dichromacy. In molecular genetics it represents the standard and simplest genotype of each dichromatic type to which other and more complex genotypes are compared [4]. This paper similarly treats the three reduction types of dichromacy generally as simple standards to which more complex variations may be compared.

It is impossible for a trichromat to know what colors a dichromat sees, but it is possible to find which chromaticities (by wavelength and purity, e.g., Figures 1 and 2) appear hued and which neutral. These chromaticities, for example confusion hues and residual hues, can only be named with certainty as they would appear to trichromats. Dichromats have learnt to use, as Dalton noted, the color names of familiar objects (sky, grass, orange fruit), sometimes without realizing their color deficiency. Despite such difficulties, estimates have been made of the residual colors actually seen by the colorblind [9]–[11]. The usual approach has been to study the colors seen by unilateral colorblinds, who have one good eye and one dichromatic [5], [12]–[16]. Such cases are rare and need careful treatment as the degree of normalcy or of dichromacy is often not soundly determined and the results may be uncertain or controversial [15], [17], [18]. From such sources it seems that both protanopes and deuteranopes see blue at about 470 nm and yellow at about 575 nm with both eyes. This corresponds well with the unique blue and unique yellow seen by color normals [19], [20]. However, this traditional position has been questioned by Mollon and Regan [21] who argue that a yellow-blue axis “cannot correspond to an axis that modulates only the short-wave cones”, and they consequently suggest residual hues of more greenish yellow and reddish blue hues. Their point is partly supported by Le Grand's [22] summary of the data on unilateral dichromats, that blue near 450 (not 470) nm and yellow near 575 nm are the only wavelengths seen by both protans and deuterans to appear the same hues with both eyes. Le Grand summarises the hues seen by tritanopes as red and greenish blue, in broad agreement with Wright [23] and Pitt [24].

Table 1 lists data on the residual hues and the confused hues for the three types of dichromacy. The table is supported by Figures 1 and 2 which illustrate the main confusion locus and minor confusion loci calculated from CIE colorimetry by Judd [25]. In the reduction form, confused hues are in complementary pairs as are residual hues. Each type of dichromacy has one neutral (or achromatic) point in the spectrum, where a monochromatic light appears neutral relative to the observer's adaptation to a given illuminant. A line from the neutral point drawn through the illuminant point to the other side of the chromaticity diagram (to what is called the confusion point, complementary to the neutral point) represents the two hues that appear neutral to the dichromat, to whom the entire line appears achromatic. Figure 1 shows other confusion loci adjacent to the main locus. The confusion loci meet at the confusion point, whose chromaticity coordinates are not exactly known but have been estimated by Pitt [24], Judd [25], and others.

**Table 1:** Characteristics of dichromacy showing wavelengths nm of neutral points and confusion points for indicated illuminant CCT, hue names of confused hues, and of residual hues.

	Reference	Obs	CCT	Neutral point	Confusion point	Confused hues	Residual hues
Protan	Judd [25]	-	6700K	493	493c	bluish green, red	blue, yellow
	Wyszecki [2]	-	6500K	490–495	493c	ditto	ditto
	Pitt [24]	5	4800K	495.5(494)	495.5c	ditto	ditto
	Hecht [28]	10	5000K	498(497)	498c	ditto	ditto
	Walls [29]	58	6500K	492	492c	ditto	ditto
	Massof [30]	9	dark	489	489c	ditto	ditto
	Sloan [32]	8	6700K	493	493c	ditto	ditto
*Mean*				***492.7(492.5)***	*492.7c*		
Deutan	Judd [25]	-	6700K	497	497c	green, bluish red	blue, yellow
	Wyszecki [2]	-	6500K	495–505	500c	ditto	ditto
	Pitt [24]	6	4800K	500.4(499)	500.4c	ditto	ditto
	Hecht [28]	12	5000K	510(509)	510c	ditto	ditto
	Walls [29]	52	6500K	498	498c	ditto	ditto
	Massof [30]	7	dark	500	500c	ditto	ditto
*Mean*				*500.2(500)*	*500.2c*		
Mean*	*less Hecht*			***498.4(498.3)***	*498.4c*		
Tritan	Judd [24]	-	6700K	567	567c/400nm	gr'sh yellow, violet	bluish green, red
	Wyszecki [2]	-	6500K	568–570	568c/447nm	ditto	ditto
	Fischer [33]	1	4800K	570(568)	570c/360nm	ditto	ditto
	Wright [23]	5	4800K	571(569)	571c/435nm	ditto	ditto
	Walls [31]	4	6500K	568	568c/445nm	ditto	ditto
*Mean*				***569(568.3)***	*569c*		

All name pairs are complementary. Means are weighted by number of observers and exclude Judd [25] and Wyszecki & Stiles [2] unsourced estimates. Mean* (under “Deutan”) denotes mean without suspect Hecht data. “Obs” denotes number of observers. Wavelengths in parentheses (including means) represent adjustment to align with illuminant CCT 6600K. Judd's neutral points are from his Fig. 2.14 (confusion loci) not Table 1.3 [24]. Under References only the 1^st^ author is listed. CCT means illuminant Correlated Color Temperature, degrees Kelvin; “dark” means dark adapted. Note Walls specified his Illum C as 6500K.

A great amount of research has gone into experimentally determining the neutral points. These are the basic and most reliable data on dichromacy. The neutral points are accurately determined by experiment for each observer, who only needs to indicate which wavelength(s) is neutral, between wavelengths that appear different hues (e.g., yellow and blue) to the observer. Table 1 lists the wavelengths of neutral points from several sources (detailed below). The wavelength's complementary according to normal trichromatic vision is listed as the confusion point. (Reductionist dichromats generally accept the color mixtures of color normals.) The names listed for confusion hues and residual hues are those used by normal trichromats and are only approximate.

The contribution and novelty of this study is the analysis of dichromats' color vision in uniform color space, CIELUV. Although dichromatic confusion loci and residual hue loci have previously been illustrated in the CIE 1931 chromaticity diagram, the latter was never intended for and is not suitable for indicating color appearance. This study is the first to illustrate and relate features of dichromatic vision in uniform color space.

## Methods and Data

The general method is colorimetry, employing both basic (e.g., color mixture) and advanced (e.g., uniform color space) colorimetry. The aim is to determine systems or interrelations in dichromacy both within and between the types of dichromacy. For those dichromats who represent the reduction form of dichromacy, their color matching functions (a pair of functions rather than a set of three) are expressed by typical or average functions calculated from the normal trichromatic functions X (λ), Y (λ), X (λ), defining the CIE standard observer as given in [2]. From such dichromatic color matching functions, the bistimulus values of protanopia, deuteranopia, and tritanopia for colors may be calculated and plotted in the CIE 1931 chromaticity diagram. Their practical use is exemplified by the protanopic, deuteranopic and tritanopic sets of confusion loci in the CIE 1931 diagram shown in Figures 1 and 2.

In Figure 1 each minor confusion locus is approximately parallel to its adjacent confusion locus, which represents a just-noticeable-difference in chromaticity from its adjacent loci. In an ideal uniform color space, the most different hues (i.e., best perceived hues) to those on the main confusion locus would be at the most different angle, i.e., orthogonal. Hues on such an orthogonal line may be expected to be the best perceived or residual hues, that is, yellow and blue in the case of red-green colorblind observers. On this basis, the residual hues are predicted in this study as lying on the orthogonal locus to the main confusion locus. However, the CIE diagram in Figure 2 was never intended for judging color appearance. The concept of orthogonality, in color appearance terms of specifying hue pairs that appear the most different from each other, requires situating within a color appearance space. In theory, the concept of orthogonality would be most accurate if the angular distribution of hues around the hue circle were perceptually uniform, in a uniform color space (UCS) or color order system as distinct from a chromaticity diagram. In the latter, the emphasis is more on basic colorimetry (e.g., color mixture) than on color appearance (e.g., color difference formulas, color discrimination ellipses). The requirement in the present paper is for a diagram that qualifies as both a chromaticity diagram (in which color mixture occurs on a straight line between color stimuli, so as to maintain exact complementarity of colors) and a uniform color space. The only system that qualifies as both is the CIELUV uniform color space.

**Figure 3:**
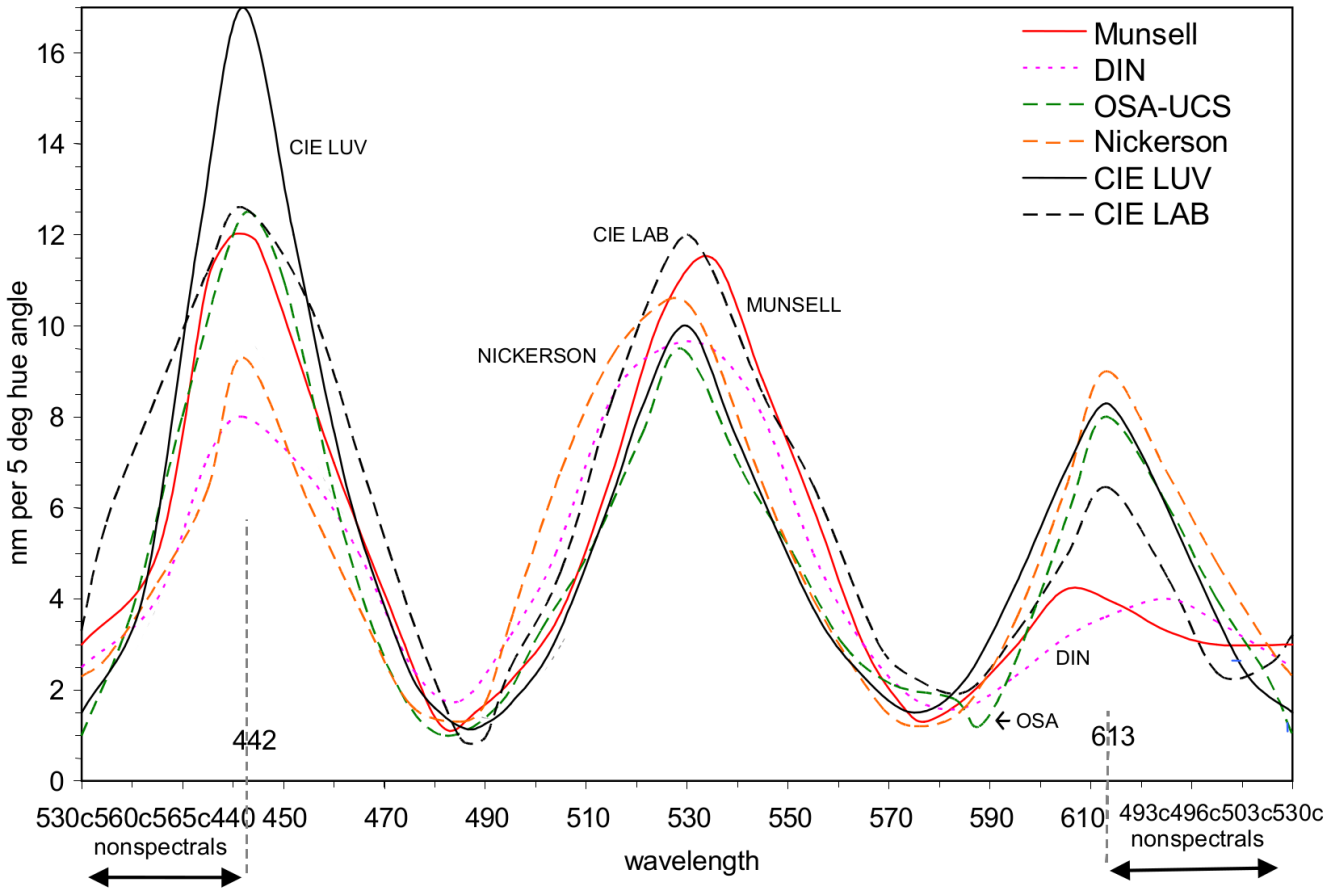
Uniform hue-difference circles for six color order systems or uniform color spaces (UCS): Munsell, DIN, OSA-UCS, Nickerson (started for OSA but never completed as a UCS), CIELUV, and CIELAB. The circles (as 360 degree hue angle circles) are compared in terms of wavelength (x-axis) and distribution of wavelength nm per 5 degree hue angle (y-axis) for monochromatic or boundary colors. The x-axis covers the full hue circle from mid-purple (530 c) through the spectrum to mid-purple again. Nonspectral hues are shown to arbitrary scale (from [26]). Dotted vertical lines at 442 and 613 nm indicate limits to the effective spectrum for monochromatic optimal color stimuli. From Pridmore [26].

Uniform (perceived) hue difference over the hue cycle is the principal basis of color order systems and color appearance spaces, of which the best known are Munsell, CIELAB, CIELUV, OSA-UCS, DIN, and Nickerson [26]. Their *uniform hue difference circles* are analyzed in Figure 3, taken from [26], compared in terms of wavelength distribution nm per 5 degrees hue angle. This figure demonstrates the well-known fact that wavelength space is highly non-uniform and does not correlate in a simple way with perceived color differences. The degree of non-uniform hue difference, represented by the curves' differences from a horizontal straight line, indicates the great difference between the CIE 1931 chromaticity diagram and uniform color space. It may be seen that the six hue circles are similar in angular distribution of wavelength though minor differences exist. For example, CIELUV is relatively higher in its short wavelength peak, while CIELAB, DIN and Munsell are lower in their long wavelength peaks. Although CIELUV is the only space that qualifies for the present study, it is clearly comparable to other UCSs or color order systems and has been used in countless color appearance studies. Incidentally, the Scandinavian NCS (Natural Colour System) does not attempt a uniform hue difference circle but for its particular purposes assumes a uniform hue angle (90 degrees) between the four unique hues Blue Green Yellow Red.

In this study, all pairs of confusion hues (and of residual hues) will be treated as exactly complementary for the following reasons. (1) The neutral point and the confusion point (i.e., the confusion hues) are necessarily complementary because a line connecting the two intersects the illuminant (white) point. (2) Only two residual hues are reportedly seen by dichromats, seen on either side of the confusion locus therefore the two hues on average are complementary. To explain further: any two wavelengths (e.g., 480 and 510 nm in deuteranopia) on opposite sides of the confusion locus appear to standard dichromats as only two hues (e.g., blue and yellow), and any two such wavelengths can admix white as is demonstrated in Figure 1, but only on the confusion locus. In terms of what the normal trichromat sees, the only exactly complementary residual hues will be those on opposite sides of the illuminant point, and these wavelengths will be acceptable to the dichromat as representing the residual hues.

Given the above conditions, the colorimetric methods to be used are CIELUV uniform color space, and complementarity and orthogonality within the uniform color space

### Basic Data

The basic data on dichromatic vision are the neutral point wavelengths, as listed in Table 1 from several sources [2], [22]–[25], [27]–[33] in three sections for the protanope, deuteranope and tritanope. Each section ends with a Mean. The wavelengths in brackets (including Means) indicate a conservative adjustment to illuminant Correlated Color Temperature (CCT) 6600K (approximating illuminants C and D65). These adjusted means were rounded to the nearest integer (493, 498, 568 nm) to be used in CIELUV diagrams in illuminant D65. Each section first lists the well known neutral point wavelengths or wavelength ranges estimated by Judd [25] and Wyszecki & Stiles [2]. Lacking explicit references and illuminants, these data cannot be included in the Mean but are useful for comparisons.

In the Deutan (also known as Deuteran) section, neutral point data from Hecht and Shlaer [28] have been criticised [28], [33] for their overly wide spread and relatively long mean wavelength (510 nm) so these controversial data are excluded from the final mean (Mean*). Some references used CIE illuminant C as standard daylight. Illuminant C has been replaced by CIE illuminant D65 (both very similar CCTs at 6740 and 6500K) as the daylight standard, but neutral points in the latter are generally the same or <1 nm shorter wavelength than Illuminant C.

For the purposes of this paper, the rounded means 493 and 498 nm are taken as neutral points for protanopia and deuteranopia and their complementaries as confusion point wavelengths in illuminant D65. For tritanopia, the neutral point and confusion point wavelengths are similarly taken as 568 nm and 568 c. These data are guides and may easily vary 1 or 2 nm between observers. Using colorimetry, Ref [34] calculated very similar neutral points for CCT 6600K at 493, 499 and 567 nm.

**Figure 4:**
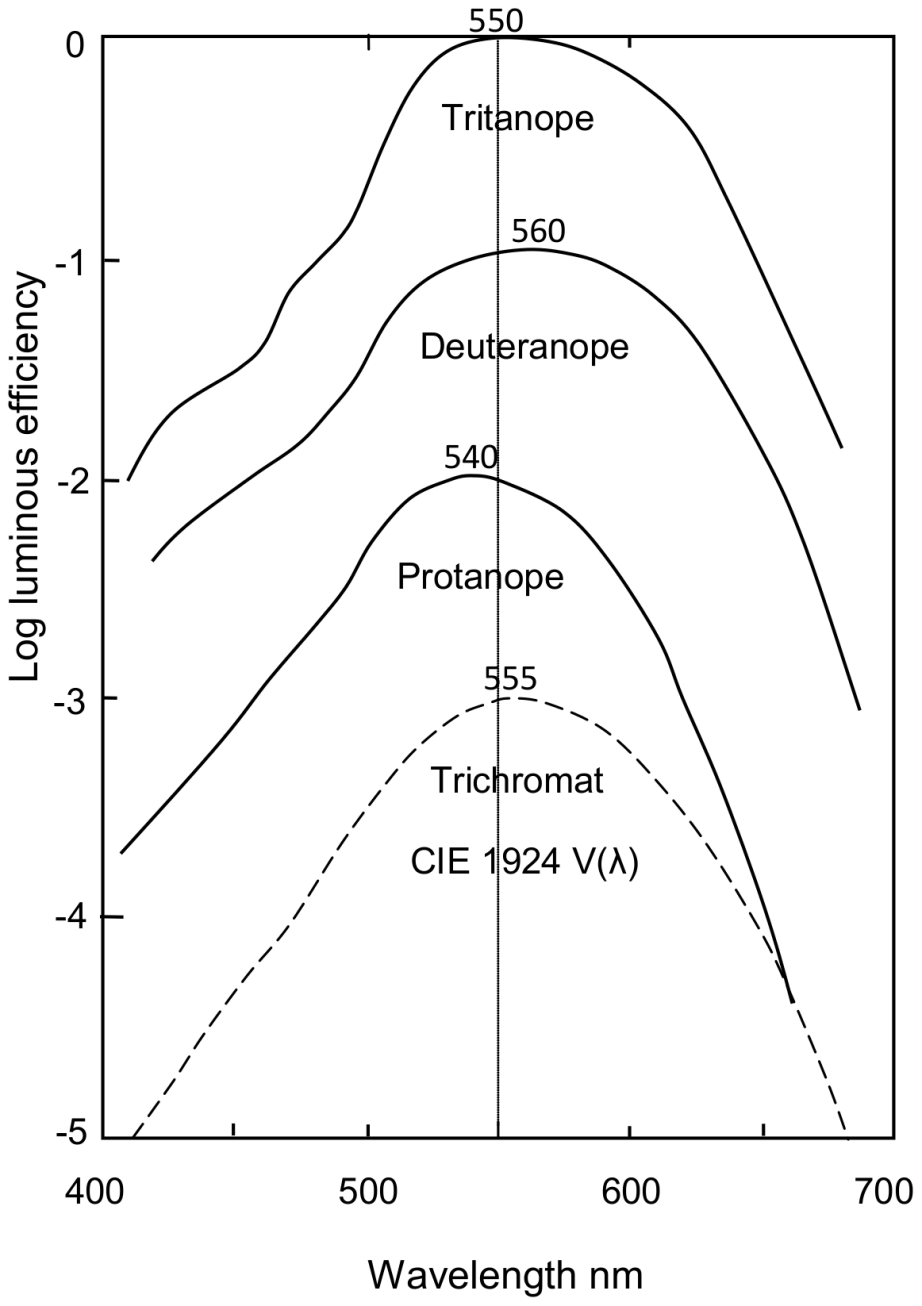
Mean luminous efficiency curves in log for protanopes, deuteranopes, tritanopes, and normal trichromats (dashed line, for CIE 1924 V(λ)). For clarity, three of the curves are displaced down by one log unit each. Redrawn from Pitt [13] and Wright [23].

**Figure 5:**
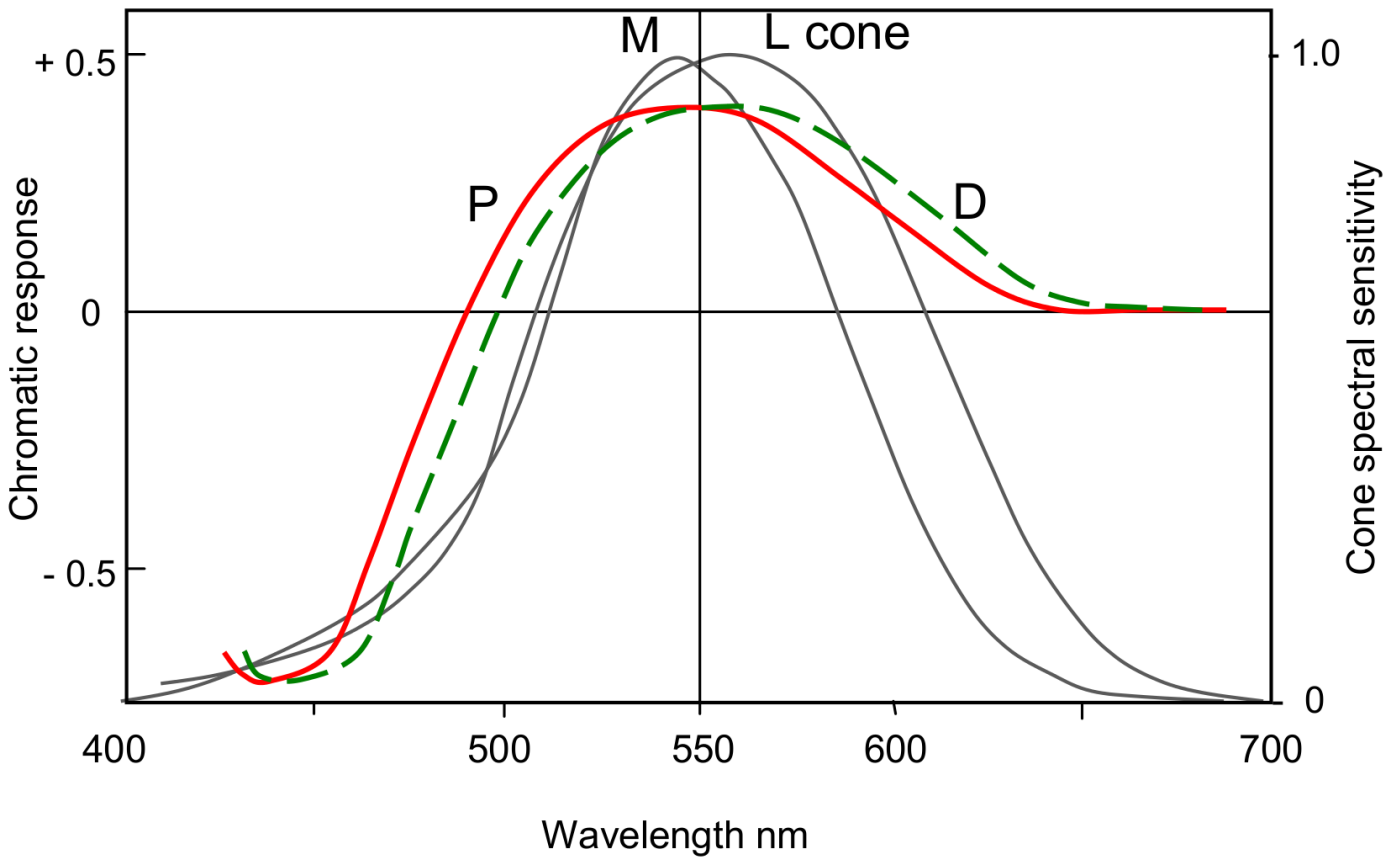
Calculated *b* and *y* chromatic response curves (*b* as negative portion, *y* as positive portion) for protanopes (P, in red) and deuteranopes (D, in green), redrawn from Hurvich & Jameson [35]. The curves intersect the zero line at 489 and 498 nm and their wavelength peaks are about 540 and 565 nm. Superimposed in gray are spectral sensitivity curves for M and L cones. In deuteranopia, yellow hues derive from the L cone curve (peak 565 nm) and its product the *y* chromatic response (see [36]), whose peak is also 565 nm. These are missing in protanopia and replaced by the M cone and its *g* chromatic response. All functions indicate shorter wavelength peaks and operating ranges for protanopia than deuteranopia.

Though the loci for protanopia and deuteranopia are similar in angle, the former's confusion loci are distinctly shorter wavelength, suggesting the protanopic residual hues are also shorter wavelength despite the convention that protanopes and deuteranopes both perceive the same hues, yellow and blue. The neutral points of protanopia and deuteranopia differ in the experimental data (Table 1) by some 5 nm or 3 just-noticeable-differences (JND). Figures 4 and 5 show important differences between protanopia and deuteranopia in their luminous efficiency curves (peaks vary from about 540 to 560 nm) and in their opponent-color *y* chromatic response curves [35].

Opponent-color chromatic responses were postulated by Hurvich and Jameson [35], supported by hue-cancellation psychophysical experiments, to represent the relative responses of blueness, yellowness, greenness, and redness (*b, y, g,* and *r*) over the spectrum. A unique hue, for example unique green, was perceivable only where the *b* and *y* curves intersected at the zero response line (i.e., were in equilibrium, see Fig. 5). The relative amplitudes of these curves at a given wavelength represented hue mixtures, say of blue-green from the *b* and *g* curves. The *b, y, g,* and *r* chromatic response curves were usually assumed (without it ever being specifically stated or demonstrated) to each represent a unique hue over all its wavelength range, as was recently confirmed in [36]. Hence these curves are also termed unique hue chromatic response curves.

As Figure 5 shows, the *y* chromatic response curves' equilibrium points for protanopia and deuteranopia vary from 489 to 498 nm, according to [35]. Superimposed in gray are the spectral sensitivity curves for M and L cones [4]. In protanopia, the L cone is not functional so the M cone and its product the *g* chromatic response curve (both peaking about 535 nm) factually substitute for the L cone and its *y* chromatic response curve in deuteranopia (and normal trichromatic vision). Hurvich and Jameson's *y* chromatic response curve (red line in Figure 5, peaking about 540 nm) for protanopia in effect represents the *g* chromatic response curve whose peak is very similar at about 535 nm [35]. Arguably therefore, Figure 5′s protanopic *y* response curve could be shifted to even shorter wavelength so its peak becomes 535 nm rather than 540.

These considerable differences indicate protanopia is strongest in luminosity and chromatic response at some 5–10 nm shorter wavelength than deuteranopia, as are the neutral point wavelengths (493, 498 nm) in Table 1. Arguably, this difference should be reflected in 5–10 nm shorter wavelength residual hues for protanopia, relative to the unique blue and yellow (about 465-480 and 570–580 nm) expected traditionally of deuteranopia. This is also the view of some others [37]–[39] who consider protanopes' residual hues are more greenish yellow and reddish blue (as judged by trichromats) than the unique yellow and blue hues seen by deuteranopes. Colorlab [39] amongst other online sites gives colored demonstrations of the different hues. Mollon and Regan advocate the same greenish yellow and reddish blue for both protanopia and deuteranopia as mentioned above and previously displayed (now removed) in Mollon's Cambridge website. Further, as Broackes notes [18] (though not himself agreeing), both Helmholtz and Maxwell in their studies of colorblindness concluded the protanope had sensations of green and violet, but called them yellow and blue and were sensitive only to yellow and blue.

As to what the deuteranope sees, it appears that the deuteranope sees only unique blue or unique yellow. With only S and L cones, and their products the *b* and *y* chromatic response curves (each of which represents one hue, a unique hue, over its entire wavelength range [36]), which nowhere overlap, there can be no mixing of *b* and *y.* Even in CIE trichromatic color mixture, there can be no mixture of unique blue and yellow (which are complementary) except to desaturate or to produce white. Further, with no M cone and no *g* chromatic response (and thus no *r* chromatic response as *g*'s balancing opponent color), there is no possibility of green or red mixing with the *b* and *y* chromatic responses, *unless* the deuteranope has some residual red-green vision (which can be the case especially in large visual fields).

The unilateral deuteranope will only see a blue or a yellow of the same hue with both eyes when that blue or yellow is the residual hue in the dichromatic eye. Whatever the latter's residual hue (and it can only be a unique blue or yellow), only that can be seen with both eyes simultaneously since it is the only hue visible in the dichromatic eye. Hence for deuteranopia, the residual hues will be unique blue and unique yellow as indeed reported in such cases.

However the protanope, with S and M cones and their product, the *b* and *g* chromatic response curves, is a more complex case. The *b* and *g* chromatic response curves do overlap [35], [36] so the protanope may possibly be seeing a mixture of unique hues. Further, it is repeatedly reported the protanopes' residual hues are blue and yellow, not blue and green, so the protanope is clearly more complex than the deuteranope (as will be described in Discussion, below).

### Orthogonal Relations

#### Daylight Illuminant D65

**Figure 6:**
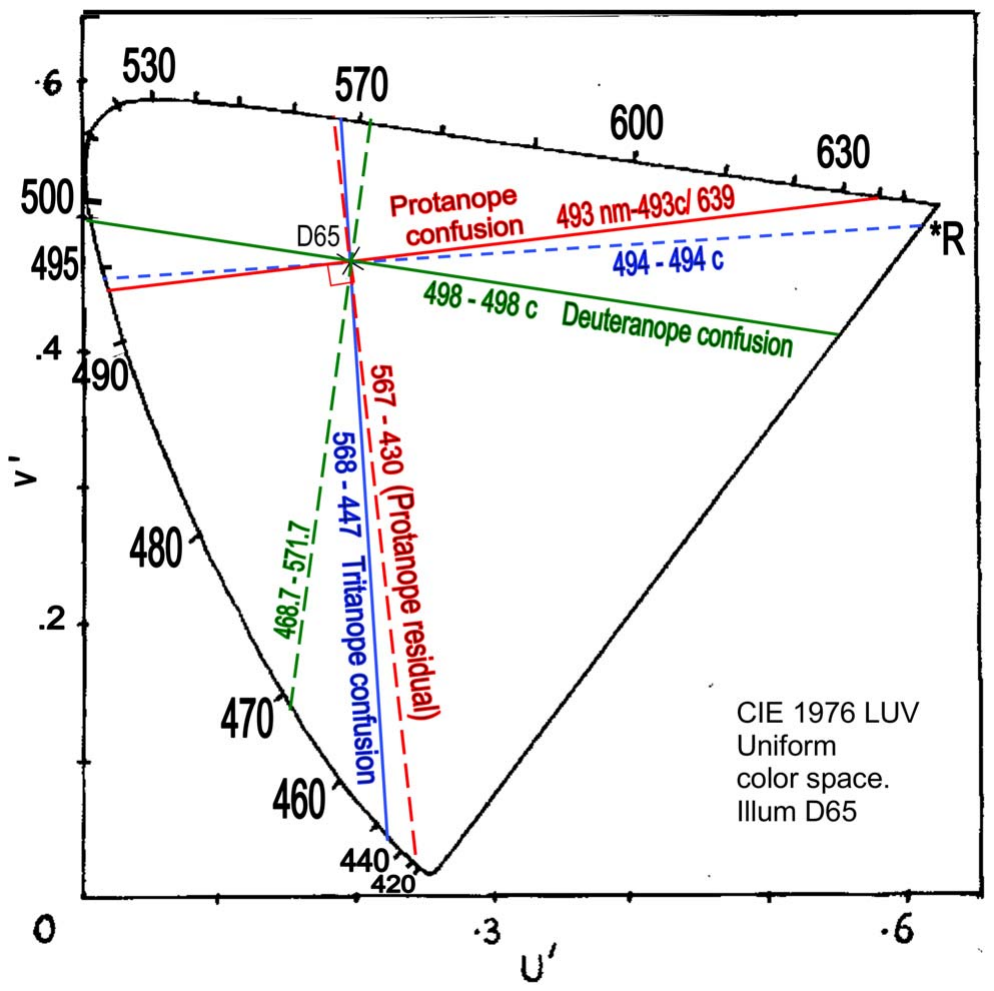
CIELUV uniform color space showing protanope (red line), deuteranope (green line), and tritanope (blue) confusion loci. The neutral point wavelengths are 493, 498 and 568 nm (Table 1), whose complementaries are the confusion point wavelengths. Orthogonal (dashed) lines indicate residual hue complementary pairs at 567–430, ca. 469–572, and 494–494 c. Illuminant is D65.

CIE 1976 LUV uniform chromaticity diagram (*aka* uniform color space) is shown in Figure 6. A uniform color space is intended to represent colors of equal difference in hue and saturation at equal distances or angles in the space, for purposes of psychological color appearance interrelations. The solid lines indicate protanopic and deuteranopic confusion loci between known wavelengths. The red line is drawn from the neutral point at 493 nm to 493 c/639 nm, which lies in or near the central range of commonly perceived unique reds, say 493–496 c [19], [20]. Exactly orthogonal dashed lines are drawn through the illuminant point to predict the two pairs of residual hues. Each line's intersection with the illuminant point means the two ends will be complementary wavelengths. The deuteranopic residual hues are indicated as about 469 and 572 nm, within the common range for unique blue and yellow as in the traditional view. The protanopic residual hues are indicated at 430 and 567 nm which represent reddish blue and greenish yellow to the trichromat. The 567 nm residual hue is some 5 nm shorter wavelength than the 572 nm residual hue for deuteranopia, as expected from the above discussion (Basic Data). In sum, orthogonality in Figure 6 has predicted the residual hues for protanopia and deuteranopia within expectations.

Confusion hues for tritanopia are plotted from 568 nm-568c/447 nm and the residual hues are predicted by drawing an orthogonal locus from 494 nm to 494 c, that is, cyan and red as expected in tradition. Surprisingly, the tritanopic residual hues locus is seen to be practically the same as the confusion locus for protanopia. One's confusion hues are the other's residual hues. Hence, protanopia and tritanopia are inverse images of each other in uniform color space, or in other words, in color appearance.

**Figure 7:**
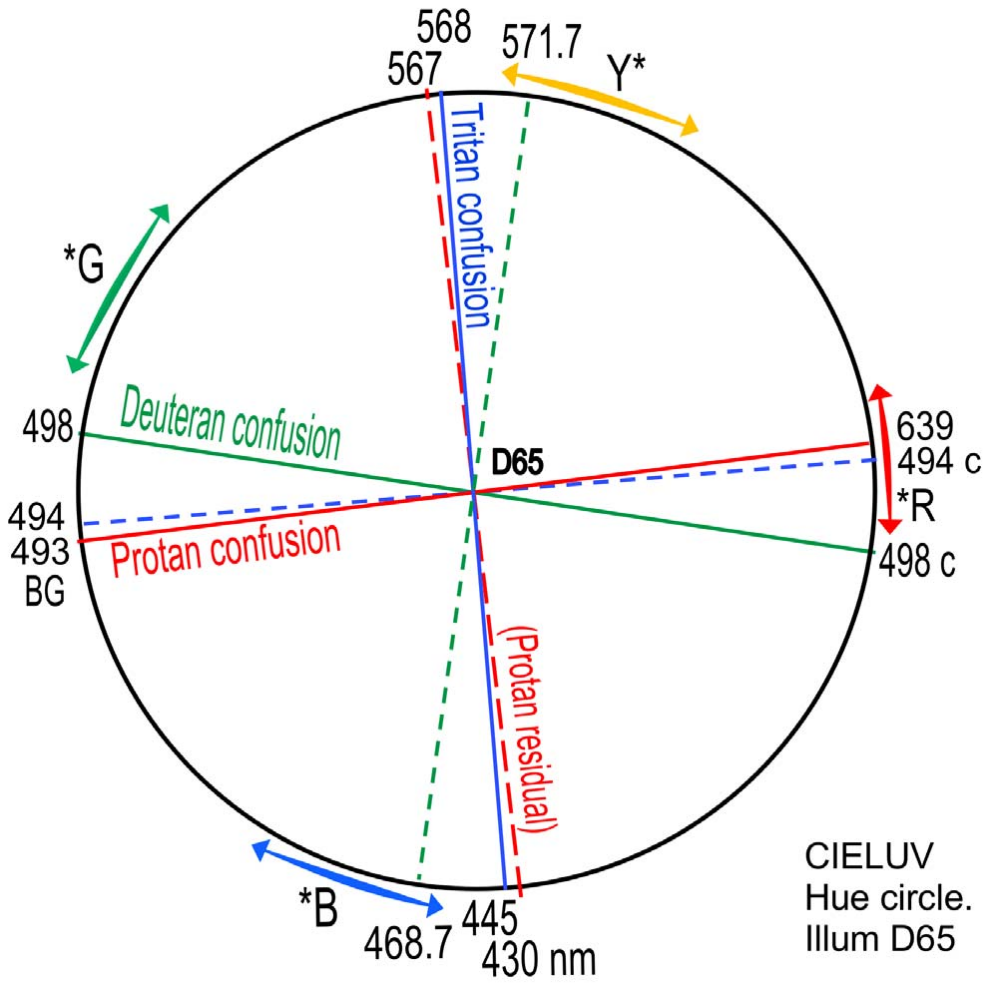
CIELUV hue circle circumscribed around the D65 illuminant point in CIELUV diagram (Fig. 6). All wavelengths retain same hue angle (from say the vertical) as in Fig 6. Indicated lines and wavelengths are transferred from Fig. 6. Common range of each unique hue (asterisks) is approximated by colored arrows outside the hue circle.

Figure 7 represents the CIELUV uniform hue difference circle (*aka* hue angle_uv_) representing a circle circumscribed around the illuminant point of the CIELUV uniform color space (Figure 6). The angles in either version of CIELUV remain identical, but Figure 7 focuses on the single dimension of hue angle rather than the two dimensions of angle and chromaticity distance in Figure 6. The latter is of course a fuller picture of the chromaticity situation but the importance of angle to the hue dimension is best illustrated in Figure 7 where all angles/ wavelengths are shown on a circle circumference equidistant to the white point at circle center. In contrast, the white point in Figure 6 is at various distances from the spectrum locus, making angles between points close to the white point seem smaller than the same angle drawn to points further from the white point.

Figures 6 and 7 show that, given complementarity and uniform color space, orthogonal relations in dichromacy exist between (1) confusion locus and residual hues locus for each type of dichromacy, and (2) protanopia and tritanopia as mutually inverse systems.

#### Munsell Hue Circle

**Figure 8:**
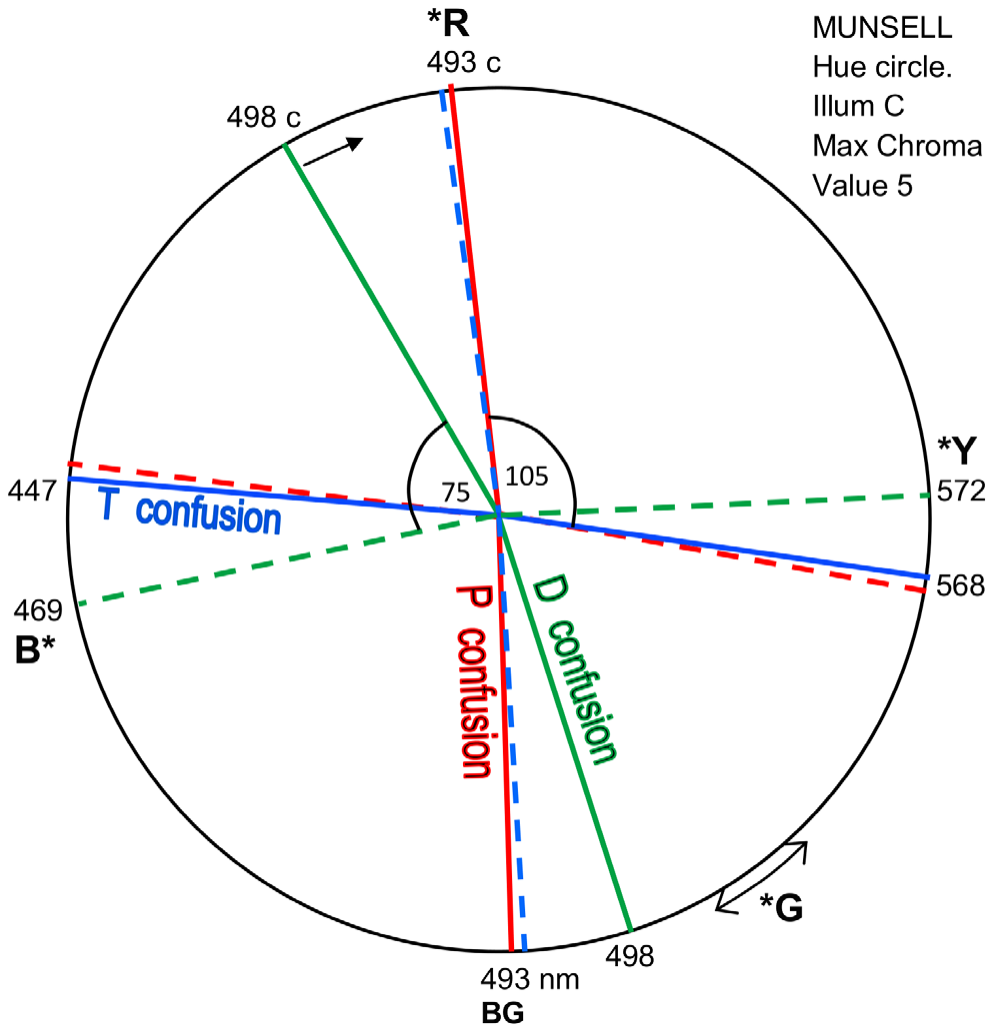
Protanopic, deuteranopic and tritanopic main confusion loci shown in Munsell hue circle for CIE Illuminant C (at Value 5 and max achievable Chroma). As in Figures 6–7, orthogonal dashed loci indicate residual hue pairs as complementary wavelengths. The inexact complementarity of Munsell space (note the indicated angles of 75 and 105 degrees which should both be 90) compromises the orthogonal relations apparent in CIELUV space (Figure 6).

The Munsell uniform hue difference circle [26] for its standard white (Illuminant C) is shown in Figure 8. It illustrates the same data as in Figure 7 but to a different hue angle scale. Munsell is based on object colors such as print colors rather than lights, and is not based on wavelengths; so these have been inserted for Value 5 (mid-luminance range) and maximum achievable saturation. This hue circle is approximately but not exactly complementary as apparent in that complementary colors such as 498 nm and 498 c are only approximately opposite through the white point.

Figure 8 indicates that orthogonal interrelations in dichromacy (Figure 7) are dependent on exact complementarity within pairs of residual hues and pairs of confusion hues. The same applies to representing the data of Figure 7 in CIE 1976 LAB color space. In both Munsell and CIELAB, orthogonal interrelations exist but the degree of exactness depends on the degree of complementarity of the respective color space.

#### Daylight (Sunlight) Illuminants B and D50

**Figure 9:**
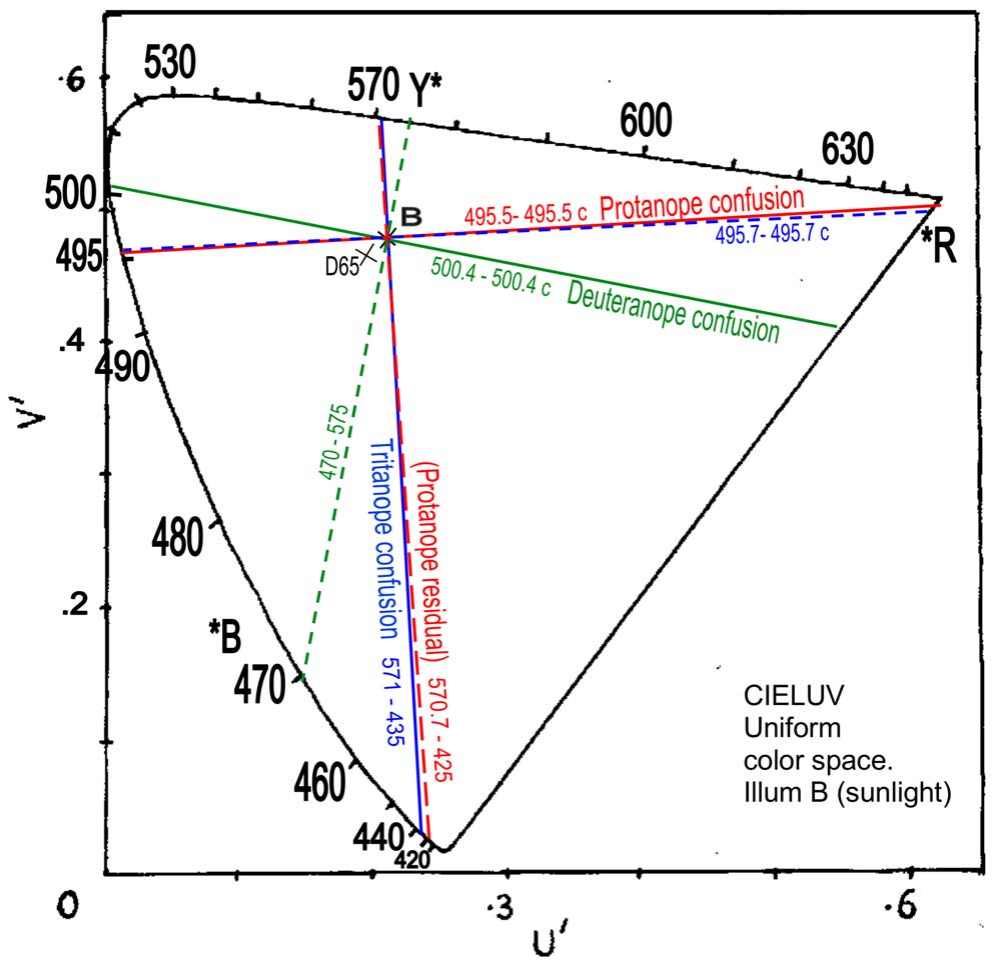
CIELUV uniform color space as in Figure 6 but for “sunlight” Illuminant B. Shows protanope confusion locus (red line) from 495.5 nm-495.5 c, and its resultant orthogonal (dashed red line) measured as 571-435 nm (predicting residual hues), deuteranope confusion locus (green line) from 500.4 nm-500.4 c and its orthogonal measured as 470–575 nm (residual hues), and tritanope confusion locus (blue line) from 571 nm-571c/435 nm and its resultant orthogonal 495.7 nm-495.7c (residual hues).

The above orthogonal relations will be checked with data in another illuminant. Some studies employed illuminants of 4800 or 5000K correlated color temperature (CCT). The most careful of such studies is by Pitt [24] whose experimental data on neutral points for protanopia and deuteranopia in illuminant B are 495.5 and 500.4 nm (Table 1). CIE illuminant B (representing sunlight) was later replaced by CIE illuminant D50 of CCT 5000K. Pitt's neutral point values are used unaltered in Figure 9 for illuminant B, which also shows orthogonal loci drawn across the confusion loci of protanopia and of deuteranopia to predict the residual hues with reasonable accuracy. Pitt did not publish data on tritanopia. Wright's [23] neutral point for tritanopia in illuminant B is 571 nm whose complementary wavelength in that illuminant is 435 nm. As Figure 9 shows, the confusion locus 571–435 nm closely aligns with the locus of residual hues for protanopia in this illuminant as in illuminant D65 (Figure 6). Besides this alignment, it is notable that the confusion locus for tritanopia is orthogonal to the confusion locus for protanopia, here and in Figure 6. This, independently of the postulated orthogonality of residual hues and confusion hues, clearly indicates orthogonal interrelations in dichromacy.

Unfortunately Figure 9 indicates problems with short wavelengths associated with the use of outdated illuminant B. Note the protanopic residual hue 425 nm and the tritanopic confusion hue 435 nm: both are shorter wavelength than the equivalents (430 and 447 nm) in illuminant D65 (Figure 6), whereas normally a hue in illuminant B or D50 is longer wavelength than in illuminant D65. Given Wright's neutral point 571 nm for tritanopia, its CIE complementary is 435 nm in illuminant B and 450 nm in illuminant D50, a substantial difference due to the especially large angle in chromaticity space between illuminant points for B and D50 relative to the spectrum region 560–580 nm.

**Figure 10:**
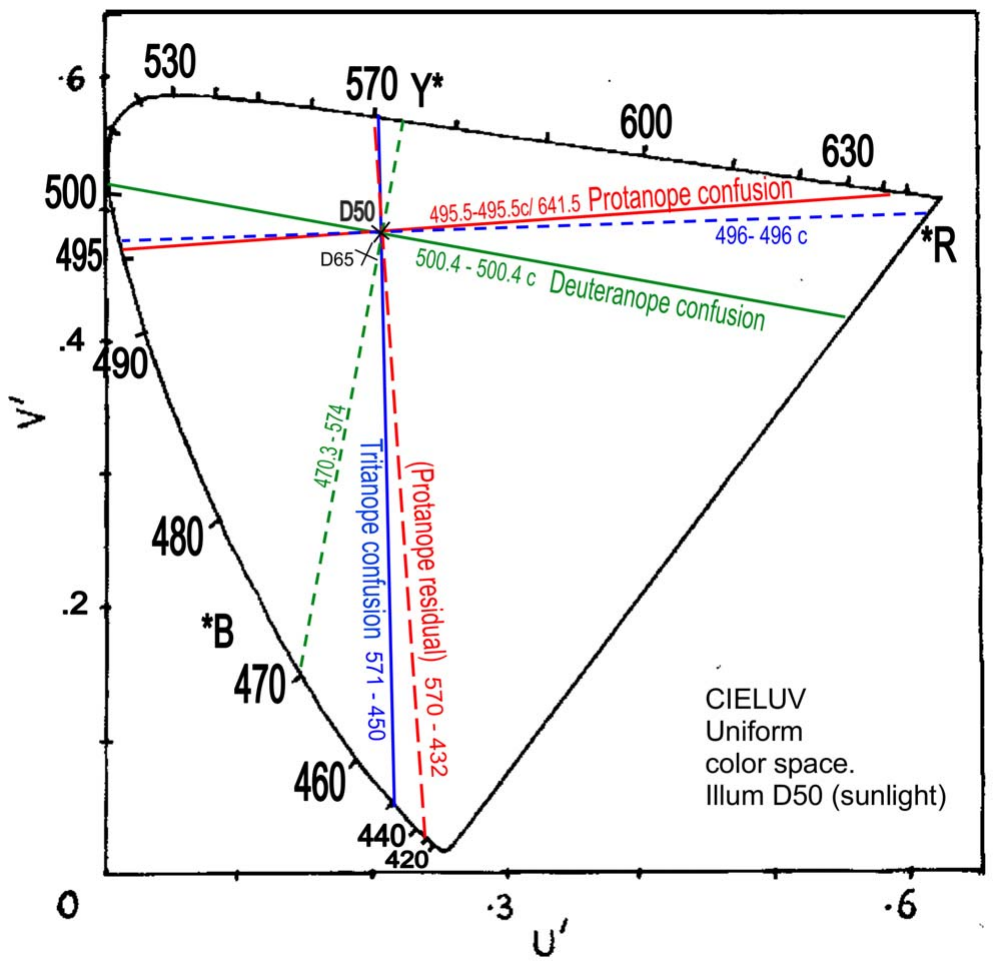
CIELUV uniform color space as in Figure 9 but for “sunlight” Illuminant D50. Shows protanope confusion locus (red line) from 495.5 nm-495.5 c, and its resultant orthogonal measured as 570–432 nm (predicting residual hues), deuteranope confusion locus (green line) from 500.4 nm-500.4 c and its orthogonal 470.3–574 nm (residual hues), and (blue line) tritanope confusion locus 571–450 nm and its orthogonal 496 nm-496 c (residual hues).

It was decided therefore to replace illuminant B in Figure 9 with the CIE replacement illuminant D50, but to make no adjustments to the experimental data. The same decision was made earlier in replacing outdated illuminant C with illuminant D65 in Figure 6. However Figure 9 was retained to record data for illuminant B, particularly the very close alignment of protanopic and tritanopic loci. Replacing both outdated illuminants C and B with the later and colorimetrically more accurate daylight illuminants D65 and D50 whilst retaining the original wavelength data for neutral points should reflect much the same angular relations in dichromacy. The complementary and other respective wavelengths were recalculated and plotted to Figure 10 as shown. Other than the short wavelengths already mentioned, wavelengths in both Figures 9A and 9B are the same to within 1 nm, and the tritanopic confusion locus remains closely aligned with the protanopic residual hues locus.

**Figure 11:**
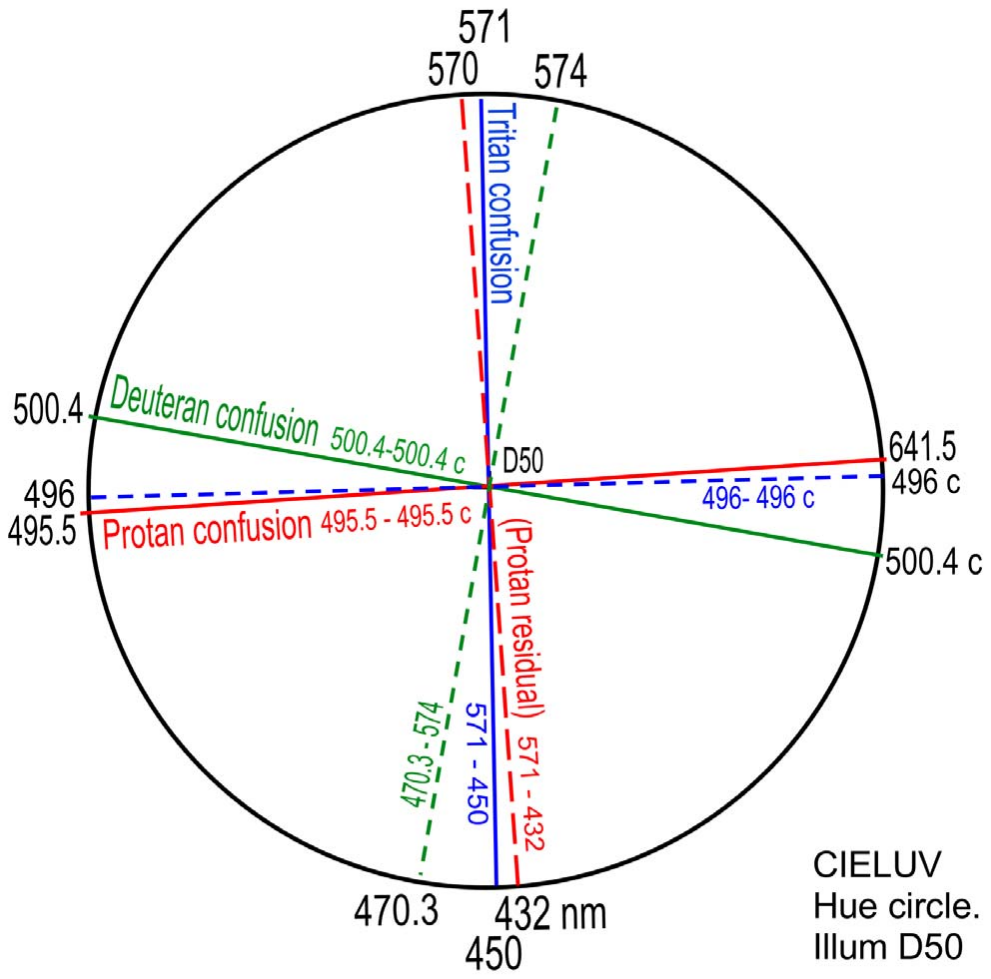
CIELUV hue circle as for Figure 7 but for Illuminant D50. Shows Protan, Deuteran, and Tritan confusion loci and their neutral point wavelengths 495.5, 500.4, 571 nm, transferred from Figure 10 CIELUV diagram, with their orthogonals (dashed lines) predicting the residual hues.

The data in Figure 10 are transferred to the CIELUV hue circle in Figure 11, with loci of course retaining the same angles around the illuminant point. Figure 11 for illuminant D50 demonstrates the same orthogonal interrelations as in Figure 7 for illuminant D65.

### Color Constancy

One might expect the color constancy judgments of a dichromatic observer to be less accurate those of a normal trichromat since the latter has additional information from a third cone. Nevertheless, studies indicate the dichromat's color constancy is generally as good as the trichromat's [40], [41].

**Figure 12:**
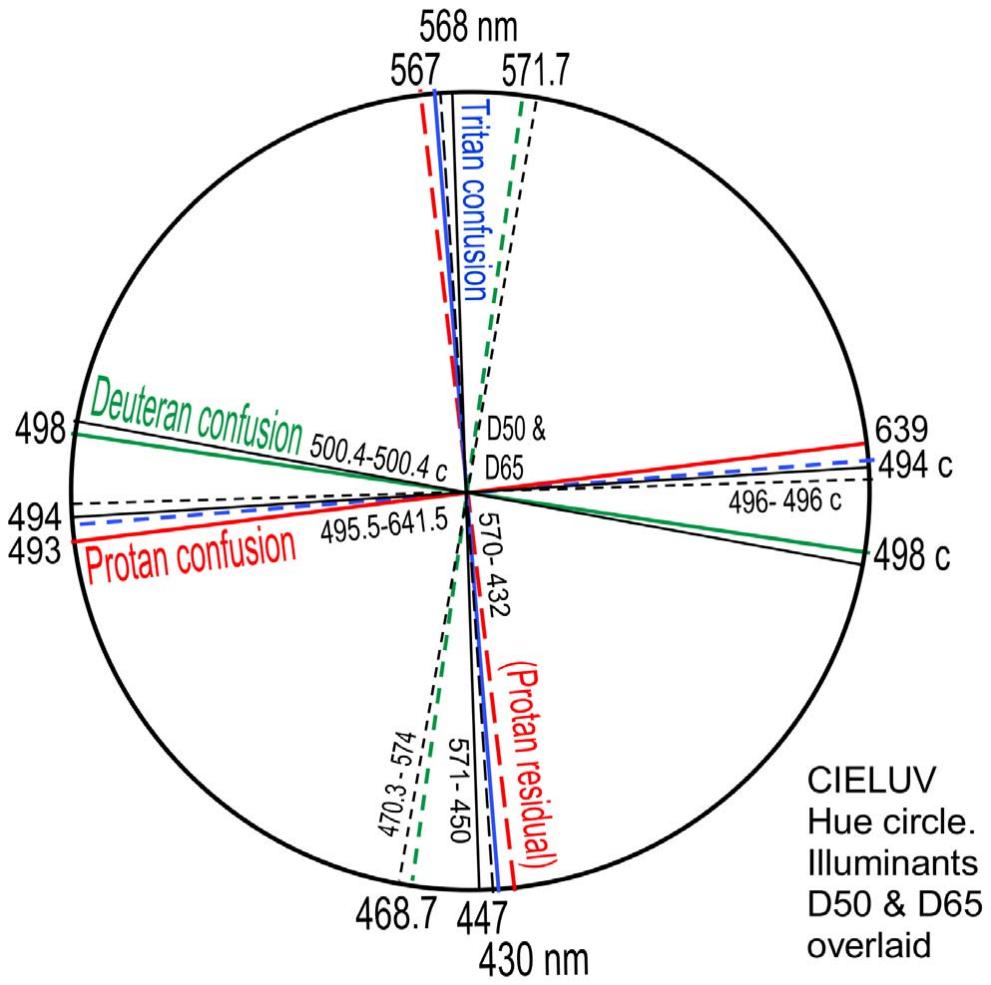
As Figure 7 (for Illum D65) but with Figure 11 (Illum D50) superimposed. The latter's lines and wavelengths are shown in black and *inside* the hue circle to compare with Figure 7′s wavelengths labeled *outside* the hue circle. Note similar angles between corresponding loci in Figures 7 and 11 despite different wavelengths, with Figure 11′s loci shifted angularly slightly clockwise relative to Figure 7′s loci, indicating chromatic adaptation.

Color constancy (the tendency of an object's color to remain approximately constant in different illuminants) is served by chromatic adaptation. The accuracy of chromatic adaption may be estimated from dichromatic data for two illuminants in this paper. Confusion loci and residual hue loci for illuminants D50 and D65 are shown in Figure 12. The figure is basically Figure 7 with loci (shown as thin black lines) superimposed. The pattern of relations in both illuminants is identical but the pattern in illuminant D50 is angularly shifted slightly clockwise. Illuminant D50 loci wavelengths (in black) are generally some 2 or 3 nm longer wavelength than the same loci for illuminant D65. For example, (1) the tritanopic neutral is 571 nm in illuminant D50 but 568 in illuminant D65; (2) the protanopic neutral is 495.5 nm in illuminant D50 but 493 in illuminant D65; (3) the deuteranopic neutral is 500.4 nm in illuminant D50 but 498 in illuminant D65.

These angle and wavelength shifts suggest chromatic adaptation. It is required to determine if the relationship between wavelengths in illuminant D65 and D50 in fact represents chromatic adaptation, and if so, how it compares with normal trichromatic vision. Pridmore [42] gives a wavelength-based chromatic adaptation method (utilising the exact wavelength shifts of CIE data on complementary colors between illuminants) that accurately predicts the wavelength shift of corresponding colors in normal vision from the following simple equation:



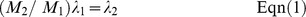
where *M* is the mean of the complementary pair of wavelengths of *minimum complementary interval (MCI)* for the respective illuminant found from a lookup table (Table II in [41]). *M_1_* (for illuminant D65) is found to be 529 nm and *M_2_* (for illuminant D50) is 531.6 nm. The terms λ_1_ and λ_2_ denote corresponding hues in illuminants 1 and 2, of which one is known and the second required. Eqn (1) works only for spectral wavelengths and not for the nonspectral complementaries/ purple hues.

The twenty available wavelengths of confusion and residual hues in illuminants D65 and D50, excluding nonspectrals, are listed in Table 2. We shall assume adaptation is from illuminant D65 to D50. Column 3 represents predicted values for D50 per Eqn (1). The Pearson correlation coefficient between columns 2 and 3 is found to be 0.99998 indicating that column 2 (for illuminant D50) represents near perfect chromatic adaptation from illuminant D65 to D50 according to normal trichromatic vision.

**Table 2:** Color constancy between illuminants D65 and D50.

Illum 65 data	Illum D50 data	Illum D50 predicted
430	432	432.1
447	450	449.2
468.7	470.3	471
493	495.5	495.4
494	496	496.4
498	500.4	500.4
567	570	569.8
568	571	570.8
571.7	574	574.5
639	641.5	642.1

Wavelength nm of confusion hues and residual hues in illuminants D65 and D50 (from Figure 12), and (in last column) D50 wavelengths predicted from the D65 data by an accurate wavelength-based chromatic adaptation model (Pridmore [42]) for normal vision. Correlation coefficient between the last two columns is 0.99998, confirming the relationship between columns 1 and 2 is near perfect chromatic adaptation according to normal trichromatic vision.

This exercise supports similar results in previous studies [40], [41] of chromatic adaptation in dichromacy. The extremely high correlation coefficient demonstrates the consistency and accuracy of the experimental data (Table 1) and the accuracy of Eqn (1). It does not of course verify the predicted residual hues, but only their chromatic adaptation from one illuminant to another.

## Results

Results for two illuminants D65 and D50 are summarized as follows:

1. Residual hue wavelengths for all three types of dichromacy were successfully predicted by an orthogonal locus to the confusion locus in uniform color space, indicating those residual hues expected from colorimetry, and unilateral dichromats; see Figures 6, 7, 9, 10.
2. The protanopic confusion locus was found to be orthogonal to the tritanopic confusion locus, and given (1) above, the protanopic system (confusion locus and residual hues locus) and the tritanopic system are reversed images of each other: One's confusion hues are the other's residual hues.
3. These orthogonal interrelations exist also in other color appearance or color order systems to the degree those systems maintain exact complementarity of wavelengths on straight lines through the illuminant point.
4. Dichromatic chromatic adaptation between illuminants D65 and D50 is demonstrated to be exactly typical of color normals.

In terms of color appearance, the residual hues were predicted and confirmed for (a) protanopia as greenish-yellow and reddish-blue, similar to some recent studies [37]–[39] but not some classical earlier studies, (b) for deuteranopia as unique yellow and unique blue in agreement with most previous studies, and (c) for tritanopia as cyan and unique red, in agreement with most previous studies. Wuerger et alia [43] also recently found that the unique red hue plane is orthogonal to the tritanopic confusion locus, by quite another method of analysis using color normal observers.

## Discussion

The above results imply four principles in the structure of dichromatic systems of the reduction or standard form: (1) complementarity of confusion hue pairs and of residual hue pairs; (2) orthogonality of confusion locus and residual hues locus within any given dichromatic system, in uniform color space; (3) orthogonality of the protanopic and tritanopic confusion loci; and (4) inverse relationship between protanopic and tritanopic systems. In the above principles, orthogonality relates to the intersection of loci at the illuminant point. Complementarity is an essential prerequisite to the above principles and to dichromatic chromatic adaptation. Without complementarity, dichromatic vision would lack structural relations, symmetry, and color constancy.

The unexpected result was finding the close inverse relationship between protanopia and tritanopia, which consists essentially of a factual orthogonal relationship between their two confusion loci. This inverse relationship might have been expected, at least approximately, since it is well known from data that tritanopia's confusion hues are cyan and red, the same as the residual hues of protanopia. The inverse relation concerns the fact that protanopia has two cones S and M while tritanopia has two cones L and M, with M in common. Hence the difference between protanopia and tritanopia rests essentially on the difference between S and L cone mechanisms. The relationship of protanopia to deuteranopia (expressed here as *P/T*) may be represented by:



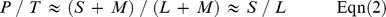
where ≈ denotes “corresponds to”. Now, S and L cone response peaks (about 445 and 565 nm [44]) are a complementary pair of wavelengths which form the near-vertical axis in Figures 7 or 10. Hence S and L share the same axis, but the axis is required by both *P* and *T. T* takes the axis as a confusion locus (as S cone wavelength peak is the neutral point) while *P* takes it as a residual hues locus (as L cone wavelength peak is a residual hue).

It is notable that in the hue circles of Figures 7 or 10, all 12 confusion hues and residual hues fall in four areas each of 15 degrees, with 90 degrees between them: a remarkably symmetrical organisation not previously noted as it only becomes apparent in the uniform hue difference circle. Two of these areas, 493–498 nm and 567–572 nm, occupy only 5 nm each, and consist of the cyan and greenish-yellow hues that in normal vision are the most discriminable in the spectrum and are the lightest of all spectrum colors. Notably the cyan region 493–498 nm contains two of the total three neutral points.

The orthogonality between these systems, each of a confusion locus and residual hues locus, systematizes their relations in color appearance space (e.g., Figures 7 or 10) and our understanding of these colorblind systems. But it does not imply these systems, as reduction forms of trichromatic vision, necessarily form parts of normal trichromatic vision. It appears true of deuteranopia, but the protanopic and tritanopic systems seem to have arisen with the deficiency, as a means of dealing with the deficiency in the manner that best ensures the residual system is visually effective.

Consider protanopia, with only two cones S and M, which directly produce the two unique hue chromatic responses *blue* and *green* as recently demonstrated [36]. The L cone is inoperative, as is its product, the unique hue chromatic response *yellow* [36] which normally opposes chromatic response *blue*. How will the visual system handle its need for complementarity, and the fact that S and M (and their resultant chromatic responses *b* and *g*) are not complementary? Either the visual system must give up its need for complementarity (which would destroy the system's ability to admix whites matching the illuminant and to maintain chromatic adaptation), or must make the unique hue chromatic responses *b* and *g* become somehow complementary. The latter is likely since we know from the above results that chromatic adaptation (which is dependent on complementary colors admixing the white of the respective illuminant) continues to be accurate in dichromacy. In that case, will S or M be the dominant cone, leaving the other cone system to behave as its complementary? As Figure 7 demonstrates, in agreement with previous researchers, the protanopic residual hues are blueish and yellowish. So it seems the S cone is dominant (as one would expect of the genetically oldest established cone in primates [45]) and has mandated the M cone and its chromatic response *g* to behave as its complementary by adopting the appearance of yellow.

In support of this argument, dichromats not only lack M or L cones or their opsins but one cone type may be replaced by the other. Hence protans not only lack L cones/ opsins but in most or many cases [5], [46] they are effectively replaced by M cones/opsins. Consequently the normal post-receptoral retinal connections to S and (the complementary) L cones are in this case between S and the replacement M cones, implying the latter instead of the (missing) L cones may now become complementaries (physiologically at least) to the S cones.

The 535 nm peak of the M cone response and also of the related *g* chromatic response curve (Figure 5) is yet another factor bringing the traditional yellow residual hue towards shorter wavelength and greener hue, as shown in Figure 7 relative to the yellow residual hue of deuteranopia.

Clearly, the protanope's perception of M cone response wavelengths about 510–560 nm (seen as green hues by color normals) as yellow is not a reduced part of normal vision but an adaptation to a genetic loss of L cone function.

It's worth noting that the importance of yellow as blue's complementary is more than just hue or wavelength. It is yellow's three-dimensional color, opposed to blue in every dimension (in its hue, low saturation and high lightness as opposed to blue's very high saturation and low lightness). Hence no other color than yellow, for example green, can complement blue. Any wavelength can complement blue so long as its color appearance (controlled by cortex) is yellow.

The above explanation of the protanope's residual (seen) hues is novel but seems satisfactory. Factually, the S cone and its chromatic response *b* continues to operate as does the M cone and its chromatic response *g*, though the latter appears definitely yellow according to unilateral protanopes. This explanation suggests that a dominant cone may account for the other dichromatic systems also. Consider tritanopia, with only the M and L cones operative. The S cone, found dominant in protanopia above, appears to be also dominant here. Its lack, and the lack of its chromatic response *b*, means its complementary yellow is also missing from tritanopia, leaving only the reddish and greenish hues shown by the experimental data in Figure 7. Hence the S cone's suggested dominance satisfactorily accounts for protanopia and tritanopia.

Consider deuteranopia, with only the S and L cones operative. The dominant S cone and its *b* chromatic response ensures that its complementary L cone also remains normally operative in both function and color appearance – yellow. This agrees with data in Figure 7 which shows residual hues of about unique blue and unique yellow. (Note the S and L cone peaks at about 445 and 565 nm [36], [44] are complementary in daylight illuminants about D65–D75.) It is worth noting that the reductionist form of deuteranopic vision appears to be the same as, or at least very similar to, early primate dichromatic vision, since both forms comprise only the S and L cones [45]. If so, it follows that deuteranopic vision is a well-developed and balanced form of color vision in itself, rather than being merely a flawed version of human trichromatic vision.

It is notable that the tritanopic confusion hue wavelengths are 445 and 568 nm, precisely the wavelength peaks of the S and L cones that comprise the deuteranopic system. Hence one might have expected 445 and 568 nm to be the residual hues of deuteranopia, as suggested by Mollon and Regan that the residual hues of red-green blindness should not correspond to the traditional yellow-blue axis but to an axis that better modulates the S cone. Such an axis is supported by this paper's preferred residual hues axis for protanopia (430–567 nm) but not for deuteranopia. The reason seems to lie again in the dominance of the ancient S cone mechanism over other cones. Of the three dichromatic types, two – tritanopia and protanopia - have reason to use their missing S or L cone response peaks as confusion hues, but only tritanopia has done so, with its missing S cone securing the S-L axis as its confusion locus. Protanopia (with its missing L cone outranked by the missing S cone of tritanopia) is thus denied the use of the S-L axis as its confusion locus, but has instead used it as its approximate residual hues locus.

It is notable that each reductionist form of dichromatism has only two residual hues. It may be expected of deuteranopia since the S and L cones directly produce the *b* and *y* chromatic response curves (unique hues blue and yellow) [35], [36], which nowhere overlap so they cannot mix any other hues such as greens. Hence the deuteranope, with only S and L cones, can theoretically perceive only the unique hues blue and yellow. This is generally supported by most investigators and unilateral dichromats. However, the situation is different for protanopia and tritanopia. Protanopia's S and M cones produce the *b* and *g* unique hue chromatic responses. Given that these chromatic responses (perceived by the protanope as blue and yellow hues) overlap in normal vision [35], [36], the question is, do the blue and yellow hues intermix to form other hues in protanopic vision? Similarly, tritanopia's M and L cones produce the *g* and *y* unique hue chromatic responses which overlap in normal vision, thus having the potential to mix intermediate hues. But apparently they do not, since most investigators agree that protanopes and tritanopes, like deuteranopes, each perceive only two residual hues.
